# Therapeutic effect of a MUC1-specific monoclonal antibody-drug conjugates against pancreatic cancer model

**DOI:** 10.1186/s12935-022-02839-w

**Published:** 2022-12-27

**Authors:** Guang Wu, Lan Li, Mengnan Liu, Chunyan Chen, Guangze Wang, Zewei Jiang, Yaqian Qin, Licai He, Hongzhi Li, Jiawei Cao, Haihua Gu

**Affiliations:** 1 Wenzhou Key Laboratory of Cancer Pathogenesis and Translation, Key Laboratory of Laboratory Medicine, School of Laboratory Medicine and Life Sciences, Ministry of Education, Wenzhou Medical University, Wenzhou, 325035 China; 2 School of Public Health and Management, Wenzhou Medical University, 325035 Wenzhou, China; 3 Medical Research Center, The First Affiliated Hospital of Wenzhou Medical University, Wenzhou, 325000 China

**Keywords:** Mucin1, Humanized monoclonal antibody, Antibody-drug conjugate, MMAE, Pancreatic cancer

## Abstract

**Background:**

Pancreatic cancer is one of the most aggressive malignancies without effective targeted therapies. MUC1 has emerged as a potential common target for cancer therapy because it is overexpressed in a variety of different cancers including the majority of pancreatic cancer. However, there are still no approved monoclonal antibody drugs targeting MUC1 have been reported. Recently, we generated a humanized MUC1 antibody (HzMUC1) specific to the interaction region between MUC1-N and MUC1-C. In this study, we generated the antibody drug conjugate (ADC) by conjugating HzMUC1 with monomethyl auristatin (MMAE), and examined the efficacy of HzMUC1-MMAE against the MUC1-positive pancreatic cancer in vitro and in vivo*.*

**Methods:**

Western blot and immunoprecipitation were used to detect MUC1 in pancreatic cancer cells. MUC1 localization in pancreatic cancer cells was determined by confocal microscopy. HzMUC1 was conjugated with the monomethyl auristatin (MMAE), generating the HzMUC1-MMAE ADC. Colony formation assay and flow cytometry were used to assess the effects of the HzMUC1-MMAE cell viability, cell cycle progression and apoptosis. Capan-2 and CFPAC-1 xenograft model were used to test the efficacy of HzMUC1-MMAE against pancreatic cancer.

**Results:**

HzMUC1 antibody binds to MUC1 on the cell surface of pancreatic cancer cells. HzMUC1-MMAE significantly inhibited cell growth by inducing G2/M cell cycle arrest and apoptosis in pancreatic cancer cells. Importantly, HzMUC1-MMAE significantly reduced the growth of pancreatic xenograft tumors by inhibiting cell proliferation and enhancing cell death.

**Conclusion:**

Our results indicate that HzMUC1-ADC is a promising novel targeted therapy for pancreatic cancer. HzMUC1-ADC should also be an effective drug for the treatment of different MUC1-positive cancers.

**Supplementary Information:**

The online version contains supplementary material available at 10.1186/s12935-022-02839-w.

## Background

Pancreatic cancer is one of the most deadly cancers, a 5-year survival of ~ 5% and a median survival of less than 11 months, a major clinical problem [1]. At present, the treatment of pancreatic cancer is limited to surgical resection and adjuvant treatments including chemotherapy and radiotherapy. The high mortality rate of pancreatic cancer means poor prognosis and lack of effective therapies [2]. Therefore, the development of novel targeted therapies for pancreatic cancer patients is particularly important.

MUC1 is a single pass type I trans-membrane glycoprotein that is with heavily glycosylated and expressed on the cell surface. MUC1 protein is auto-proteolycally cleaved at the GSVVV motif, located within the sea-urchin sperm protein, enterokinase and agrin (SEA) domain, generating two peptide fragments of the MUC1-N and MUC1-C subunits. The two subunits form heterodimers through non-covalent bonds [3]. MUC1-N is composed of variable tandem repeat region (VNTR) and SEA domain. The VNTR region is composed of 20 amino acids that are extensively O-linked glycosylated at the serine and threonine residues. MUC1-N and MUC1-C are sparingly N-linked glycosylated at asparagine residues [4]. MUC1-C contains a short extracellular region, transmembrane domain, and a cytoplasmic tail.

MUC1 is mainly located in the apical membrane of epithelial cells. In a variety of epithelial cancers including breast cancer and pancreatic cancer, MUC1 is often abnormally over-expressed and evenly distributed on the entire surface of cancer cells due to the loss of polar expression. Therefore, it is one of the important targets for cancer therapy [5–11]. More than 90% of pancreatic cancers have abnormally high expression of MUC1. The intracellular region of MUC1-C plays a key role in the growth and metastasis of pancreatic cancer cells. MUC1-C associates with HIF-α and promotes its translocation onto the nucleus, resulting in the increased production and secretion of PDGFA, which interacts with the receptor PDGFR-α signaling through PDGFR-α has an additive effect on β-catenin translocation, enhancing MUC1-C induced proliferation and invasion of pancreatic cancer cells [12].

Currently, there is no antibody drug targeting MUC1 approved by the U.S. Food and Drug Administration (FDA). The main reason is that almost all MUC1 monoclonal antibodies used in clinical research and development target the high immunogenic VNTR in MUC1-N [13]. These anti-MUC1-N antibodies recognize either the non-glycosylated polypeptide portion [14] or the glycosylated chain of VNTR or simultaneously recognize non-glycosylated peptide and glycosylated chain of VNTR [15–17]. However, all these MUC1 antibodies that only recognize the epitopes in the MUC1-N subunit are not effective in clinical trials [17]. One of the main reasons is that there are large amounts of MUC1-N shedding from the surface of tumor cells in cancer patients [18]. The free MUC1-N subunit may neutralize most MUC1 therapeutic antibodies, which limits the amount of antibodies targeting MUC1 protein on the surface of tumor cells [13, 18, 19].

We recently generated a novel mouse monoclonal antibody specifically recognizing the human MUC1 SEA domain (MUC1-SEA Ab) with some inhibitory efficacy against pancreatic cancer in xenograft model [20]. Furthermore, we developed a novel humanized MUC1 antibody (HzMUC1) from MUC1-SEA Ab, which specifically recognizes the interaction region between MUC1-N and MUC1-C in its native state [21]. HzMUC1 was used to generate antibody drug conjugate (ADC) through conjugation with the antineoplastic agent monomethyl auristatin (MMAE). HzMUC1-MMAE was found to significantly inhibit the growth of MUC1-positive trastuzumab-resistant HER2 positive breast tumors [21]. In this study, we evaluated the effects of HzMUC1-ADC against pancreatic cancer cells in vitro and in vivo. Our result shows treatment with HzMUC1-MMAE is a potential therapy for the treatment of pancreatic cancer.

## Methods

### Antibodies

The humanized MUC1 antibody (HzMUC1) was generated as described previously [21]. Briefly, to obtain the humanized IgG1 antibody with intact IgG format, DNAs encoding VH and VL were synthesized (Tsingke, China) and inserted into the modified pcDNA 3.4 expression vector (Thermo Fisher Scientific, Waltham, MA, USA) carrying the human IgG1 constant region (CH1-hinge-CH2-CH3) or the human kappa chain constant region (CL). These vectors were transiently co-transfected into HEK-293 F cells. After 6 days, HzMUC1 antibody was purified from culture supernatants using Protein-A chromatography (GE Healthcare Life Sciences, Buckinghamshire, UK).

To detect MUC1 protein in cells by western blotting and immunoprecipitation, commercially available rabbit monoclonal antibody against the cytoplasmic tail of MUC1 (CT) (anti-MUC1-CT Ab, Catalog No. ab109185 at 1:5000) was purchased from Abcam (Cambridge, UK). Anti-MUC1 mouse monoclonal antibody to MUC1-N (clone GP1.4; Catalog No. AM32842PU-S at 1:2000) was obtained from OriGene (Rockville, USA). The anti-β-actin antibody (Catalog No. 3700 S at 1:5000), anti-Ki-67 antibody (Catalog No. 9027 S at 1:400), anti-cleaved caspase-3 antibody (Catalog No. 9661 S at 1:1000), anti-caspase-3 antibody (Catalog No. 9662 S at 1:1000) were purchased from Cell Signaling (Danvers, MA, USA).

### Cell culture

Human pancreatic cancer cell lines Capan-2, CFPAC-1, SW1990, Mia-PaCa-2, PATU-8988, PANC-1, and Normal pancreatic duct cell line hTERT-HPNE were purchased from American Type Culture Collection (ATCC, Manassas, VA, USA). Capan-2 cells were cultured in McCoy’s 5 A medium (Thermo Fisher Scientific). CFPAC-1 cells were cultured in Iscove’s modified Dulbecco’s medium (IMDM, Thermo Fisher Scientific). SW1990, Mia-PaCa-2, PATU-8988, PANC-1, and hTERT-HPNE cells were cultured in Dulbecco’s modified Eagle’s medium (DMEM, Thermo Fisher Scientific). All media were supplemented with 10% fetal bovine serum (Thermo Fisher Scientific), penicillin (100 U/mL), and streptomycin (100 µg/mL). Cells were incubated at 37 °C in an atmosphere of 5% CO_2_. HEK-293 F cells were cultured in suspension with serum-free medium (Catalog No. 12338018; Thermo Fisher Scientific), and incubated at 37 °C in an atmosphere of 8% CO_2_.

### Immunoblotting and immunoprecipitation

For immunoblotting, cell lysates were boiled and separated by SDS-PAGE, transferred onto PVDF membranes (Millipore, Billerica, MA, USA), and blocked with 5% non fat skim milk in TBS-T for 1 h at room temperature. The membranes were probed with primary and HRP-conjugated secondary antibodies (Jackson ImmunoResearch Laboratories, West Grove, PA, USA), and developed using chemiluminescence (ECL) reagent. The immunoreactive proteins were detected using ChemiDoc MP imaging system (Bio-Rad) and analyzed with Image Lab 5.0 software (Bio-Rad).

For immunoprecipitation, cells were lysed in 1% NP-40 lysis buffer containing protease inhibitor cocktail mixtures (Selleck Biosciences, San Jose, CA). Cell lysates were incubated with HzMUC1 antibody overnight at 4ºC and followed by incubation with Protein A bead for an additional 1 h. The immunocomplexes were washed with lysis buffer and denatured with Laemmle buffer, followed by immunoblotting with anti-MUC1-CT antibody as described previously.

### Confocal microscopy

Pancreatic cancer cells were seeded on poly-L-lysine-coated glass coverslips in 24-well culture plates. After 72 h, cells were blocked with 3% BSA, and incubated with HzMUC1 antibody for 1 h on ice, washed with PBS containing 1% BSA, and incubated with Alexa Flour 488-conjugated goat anti-human IgG antibody (Jackson ImmunoResearch) for 30 min. All samples were mounted and observed under the Nikon A1 confocal microscope system (Nikon, Tokyo, Japan).

### Generation of antibody-drug conjugate

The HzMUC1-MMAE and human IgG-MMAE ADC were generated as described previously [21]. Briefly, HzMUC1 or human IgG (Bethyl Laboratories, Montgomery, TX, USA) were partially reduced with tris (2-carboxyethyl) phosphine hydrochloride (TCEP, Sigma-Aldrich St. Louis, USA) at 37 °C for 2 h, and the buffer was exchanged by passing the mixture through Sephadex G25 resin and eluted with PBS. The drug-linker agent (mc-vc-PAB-MMAE, MCE, Monmouth, NJ, USA) dissolved in DMSO was then added to the reduced antibody at 4 °C for 1 h. The reaction mixture was concentrated by centrifugal ultra-filtration, passed through Sephadex G25, and eluted. The eluate was then sterile filtered through a 0.2-µm filter and stored 4 °C.

### Colony formation assay

Cells were seeded in 48-well tissue culture plates (3 × 10^3^ cells/well), and treated with the indicated concentrations of HzMUC1 antibody, human IgG-MMAE, and HzMUC1-MMAE. After 5 days, colonies were fixed with 4% paraformaldehyde solution, and stained with 0.5% crystal violet solution at RT for 20 min. Stained crystal violet dye was solubilized with 10% acetic acid. Absorbance was measured at 540 nm using a microplate reader (Molecular Devices, San Jose, CA). The inhibitory concentration (IC_50_) for HzMUC1-MMAE was determined using SPSS statistics software.

### Cell cycle analysis

Pancreatic cancer cells were treated with HzMUC1-MMAE for 24 h, harvested by trypsinization, washed with cold PBS, and fixed with cold 70% ethanol at 4 °C for 2 h. The fixed cells were washed with PBS and incubated with Propidium iodide (Dojindo, Kumamoto, Japan) at 37 °C in the dark for 30 min and detected by Flow cytometry (NovoCyte™, ACEA Biosciences, Hangzhou, China) and analyzed using the NovoExpress software.

### Apoptosis assay

Cells were seeded in 6-well culture plates, and treated with the indicated concentrations of human IgG-MMAE and HzMUC1-MMAE. After 72 h, cells were harvested with trypsin-EDTA and washed with FACS buffer. The apoptotic cells were stained with annexin V and propidium iodide (Dojindo) for 15 min at RT in the dark, and analyzed via Flow cytometry (NovoCyte™, ACEA Biosciences) using the NovoExpress software.

### Xenograft tumor studies

The experimental protocol was approved by the by the Institutional Animal Care and Use Committee of Wenzhou Medical University (Permit Number: 2020-015). Capan-2 cells (3.5 × 10^6^) or CFPAC-1 cells (3 × 10^6^) were resuspended in 50% Matrigel (Corning, Bedford, MA, USA) and injected subcutaneously into the dorsal right flank of six-week old female BALB/c nu/nu mice (purchased from GemPharmatech, Jiangsu, China) .When the tumors grew up to about the size of 120 ~ 150 mm^3^, mice were randomly divided into two groups, PBS and HzMUC1-MMAE (5 mg/kg). PBS or ADCs were injected intravenously into the mice every six days for three times. Tumor diameters were measured with calipers, and tumor volumes were calculated using the formula, *V* = width^2^ × length/2. Mice were sacrificed on the 18th (Capan-2 tumor) or 21st day (CFPAC-1 tumor) after ADCs injection. On day 18 ~ 21 after ADCs injection, all mice were euthanized, and the tumors were surgically excised, weighed, fixed in 10% formalin, and embedded in paraffin.

### Immunohistochemistry

Paraffin blocks of tumor were sliced into 4 μm-thick sections and dried onto slides. The sections were deparaffinized, rehydrated, and treated with 3% hydrogen peroxide for 30 min. For antigen retrieval, slides were immersed in citrate solution (pH 6.0) heated at 121 °C for 20 min, and then blocked with 3% normal horse serum for 30 min. The slides were incubated with anti-Ki-67 antibody (1:400) or anti-cleaved caspase-3 antibody (1:100) overnight at 4 °C followed by incubated with HRP-conjugated anti-rabbit IgG antibody (ZSGB-BIO, Beijing, China) for 1 h. The immunoreactivities were detected with 3,3′-diaminobenzidine (DAB, ZSGB-BIO) and examined under an Eclipse Ci microscope (Nikon, Japan).

### Statistical analysis

All data are presented as mean ± standard deviation. Statistical significance of differences between two samples was performed using unpaired two-tailed Student’s *t*-test. One-way ANOVA with Bonferroni’s multiple comparison test correction was used to analyze data among multiple groups. Statistics were performed using Prism (Graph Pad Software Inc). Statistically significance was considered when *p*-value was less than 0.05. All experiments were repeated at least three times.

## Results

### HzMUC1 antibody binds to MUC1 on the cell surface of pancreatic cancer cells

**Fig. 1:**
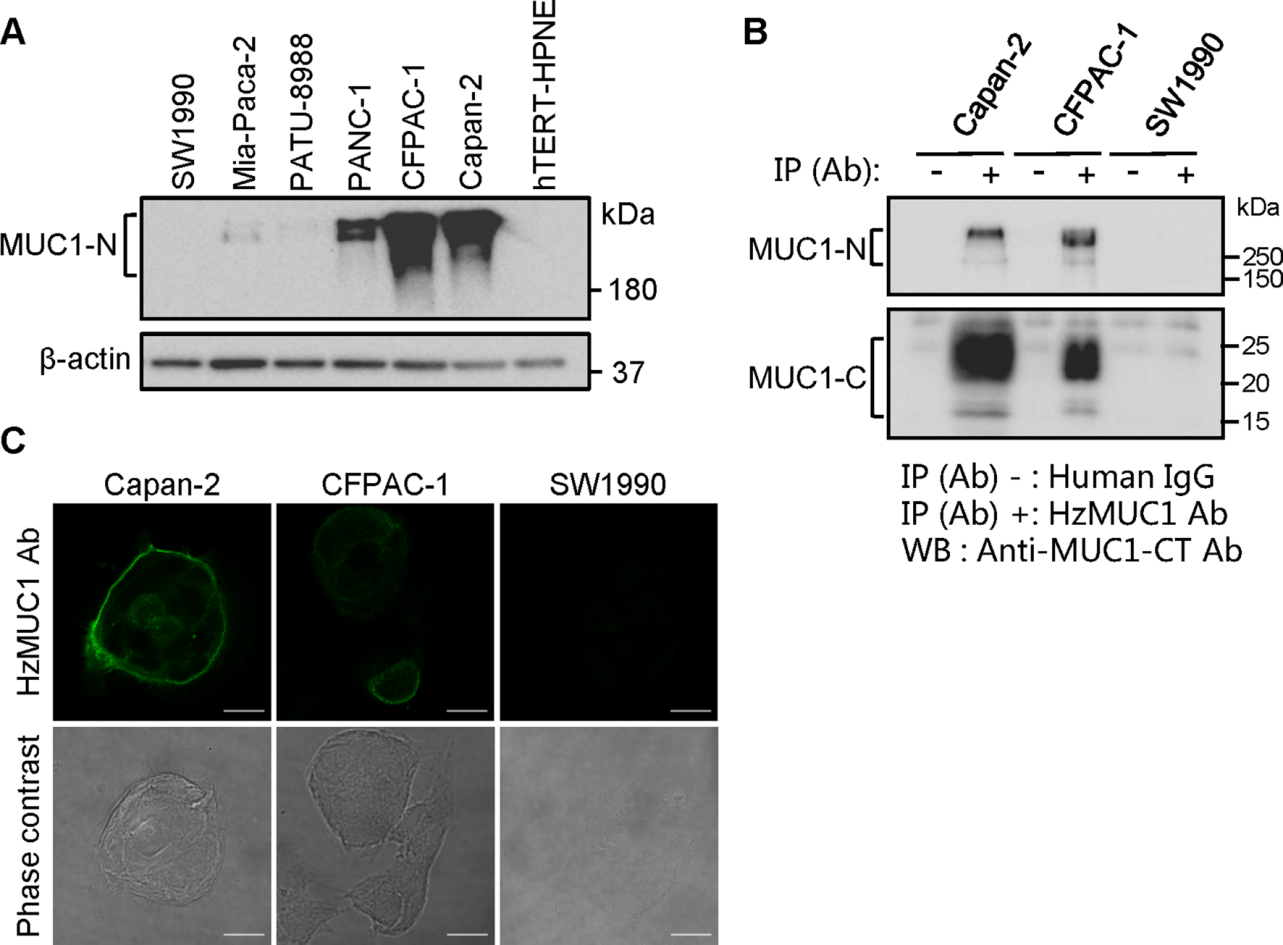
HzMUC1 antibody binds to MUC1 on the cell surface of pancreatic cancer cells. **A** Lysates from SW1990, Mia-PaCa-2, PATU-8988, PANC-1, CFPAC-1, and Capan-2 pancreatic cancer cell lines, and hTERT-HPNE (a normal epithelial pancreatic cell line) were resolved by SDS-PAGE and immunoblotted with the commercially available anti-MUC1 N-terminal (NT) antibody and reprobed with anti-β-actin antibody as loading control. **B** Cell lysates from Capan-2, CFPAC-1, and SW1990 cells were immunoprecipitated with human IgG or HzMUC1 antibody and immunoblotted with the anti-MUC1-NT antibody or anti-MUC1-CT antibody. **C** HzMUC1 antibody binds to MUC1 protein on the cell surface. Capan-2, CFPAC-1 and SW1990 cells grown on coverslips were washed with PBS, incubated with HzMUC1 antibody (1 µg/well) on ice, and subsequently with Alexa 488-conjugated secondary antibody, and examined with fluorescent (upper panels) and phase contrast (lower panels) confocal microscopy. Scale bars, 20 μm

To examine if the HzMUC1 antibody recognizes MUC1 protein in pancreatic cancer cells, we performed western blot analysis with lysates from Capan-2, CFPAC-1, PANC-1, Mia-PaCa-2, PATU-8988, SW1990 cells, and hTERT-HPNE cells. The commercial anti-MUC1-NT antibody (epitope: DTRP in the tandem repeats) detected MUC1-N protein in pancreatic cancer cells, with the highest expression of MUC1-N in Capan-2 and CFPAC-1, followed by PANC-1 cells, and the lower extend in Mia-Paca-2 and PATU-8988, but not detected in SW1990 and Normal pancreatic duct cell line hTERT-HPNE cells. We therefore used the SW1990 cell line as a negative control throughout this study (Fig. 1A).

To evaluate whether the HzMUC1 antibody recognizes endogenous MUC1 in the native state in cells, HzMUC1 antibody was used to immunoprecipitate MUC1 followed by immunoblot analysis in the above three cell lines. The result showed that HzMUC1 immunoprecipitated the native MUC1 proteins in Capan-2 and CFPAC-1 cells (Fig. 1B). The amount of immunoprecipitated MUC1-N in Capan-2 cells was similar to that in CFPAC-1 cells. In contrast, the amount of immunoprecipitated MUC1-C in Capan-2 cells was about ~ 3 fold higher than that in CFPAC-1 cells. Furthermore, these cells were immunostained with HzMUC1 without permeabilization and fixation. Consistently with the immunoprecipitation result, the immunofluorescent staining signals were significantly higher on the cell surface of Capan-2 cells than that of CFPAC-1 cells, whereas immunofluorescent staining signal was detected in SW1990 cells (Fig. 1C). These results indicate that the HzMUC1 antibody efficiently recognizes the native form of MUC1 protein in and on the surface of MUC1 + pancreatic cancer cells.

### HzMUC1-MMAE inhibit the growth of MUC1 positive pancreatic cancer cells

**Fig. 2:**
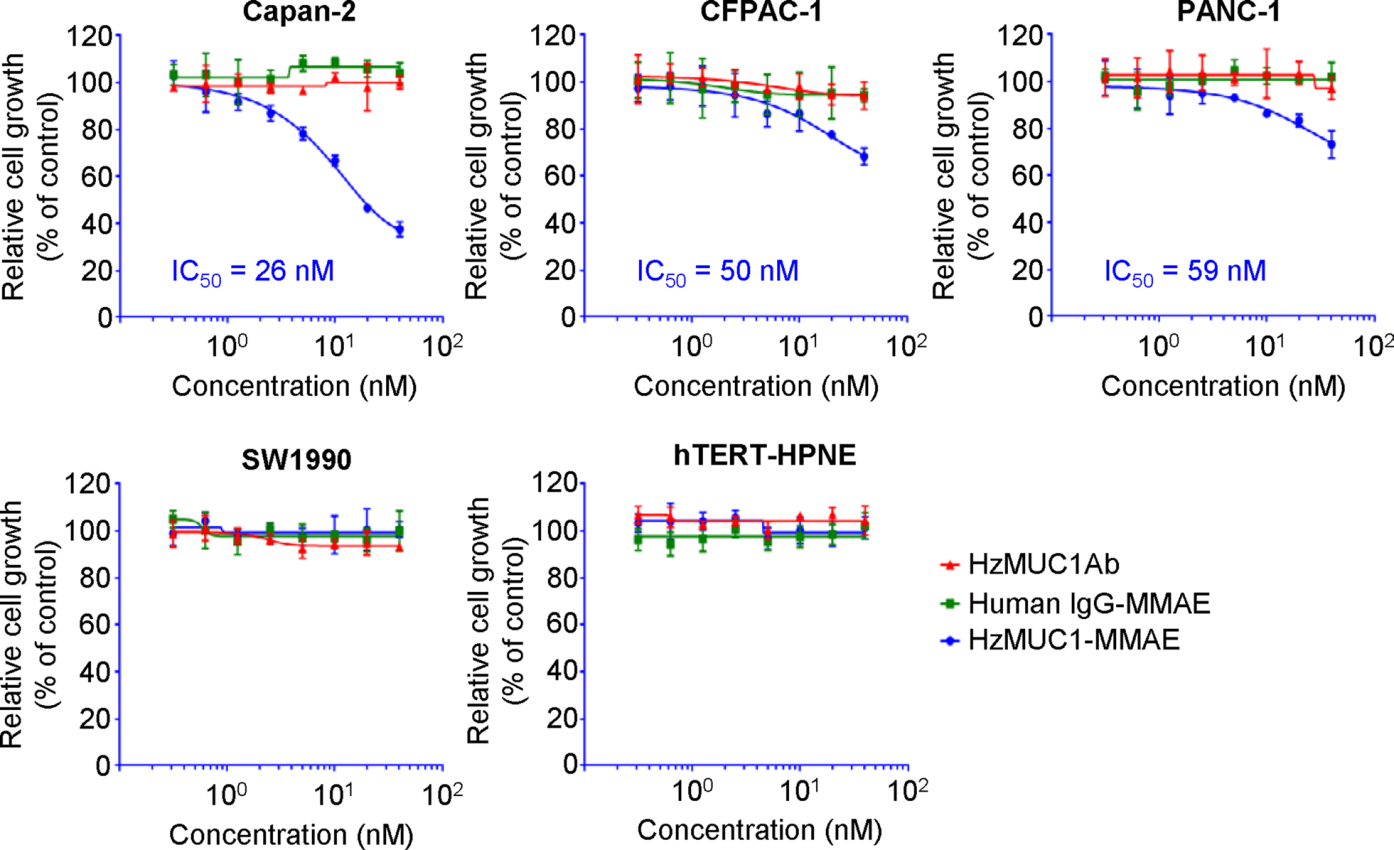
HzMUC1-MMAE inhibits the growth of pancreatic cancer cells. Capan-2, CFPAC-1, PANC-1, SW1990, and hTERT-HPNE cells were plated at low cell density and incubated with indicated concentrations of HzMUC1 antibody, human IgG-MMAE, and HzMUC1-MMAE for 5 days. Cell growth was determined by staining the plates with crystal violet and quantified by OD 540 nm absorbance. Percent of relative cell growth was shown as the non antibody treatment was set to 100. The IC_50_ values are indicated. The results shown are representative of data from 3 independent experiments

We investigated the efficacy of HzMUC1 antibody conjugated with chemotherapeutic drugs on pancreatic cancer cells. Firstly, we used valine-citrulline dipeptide as linker to conjugate HzMUC1 antibody with monomethyl auristatin E (MMAE), a microtubule polymerization inhibitor, generating the antibody drug conjugate, HzMUC1-MMAE. Then, we examined whether HzMUC1-MMAE can inhibit the growth of pancreatic cancer cells. MUC1 positive (Capan-2, CFPAC-1, PANC-1), MUC1 negative (SW1990) pancreatic cancer lines and Normal pancreatic duct cell line hTERT-HPNE were treated with different concentrations of human IgG-MMAE, HzMUC1-MMAE and analyzed using colony formation assay. As an isotype control, human IgG-MMAE did not have significant effect on the growth of Capan-2, CFPAC-1, PANC1, SW1990, and hTERT-HPNE cells. In contrast, compared with human IgG-MMAE, HzMUC1-MMAE significantly reduced the proliferation of Capan-2 cells (IC_50_ = 26 nM), CFPAC-1 (IC_50_ = 50 nM) and PANC1 (IC_50_ = 59 nM). HzMUC1-MMAE was most sensitive to Capan-2 cells followed by CFPAC-1 and PANC-1 cells (Fig. 2). HzMUC1-MMAE did not have significant effect on the growth of MUC1 negative cells SW1990.

In addition, we tested the effect of HzMUC1 antibody, drug-linker agent (Mc-vc-PABC) and MMAE separately on cell proliferation of the four cell lines mentioned above. HzMUC1 antibody and drug-linker agent could not effectively inhibit the growth of cells (Fig. 2, Additional file 1: Figures S1, S2). In contrast, MMAE alone inhibited the growth of Capan-2, CFPAC-1, and hTERT-HPNE cells, with IC_50_ of 108 nM, 79 nM, and 86 nM, respectively, and did not affect the growth of SW1990 cells (Fig. 2, Additional file 1: Figures S1, S2). Our results indicate that HzMUC1-MMAE can inhibit the growth of MUC1 positive pancreatic cancer cells.

### HzMUC1-MMAE induces G2/M cell cycle arrest of MUC1 positive pancreatic cancer cells

**Fig. 3:**
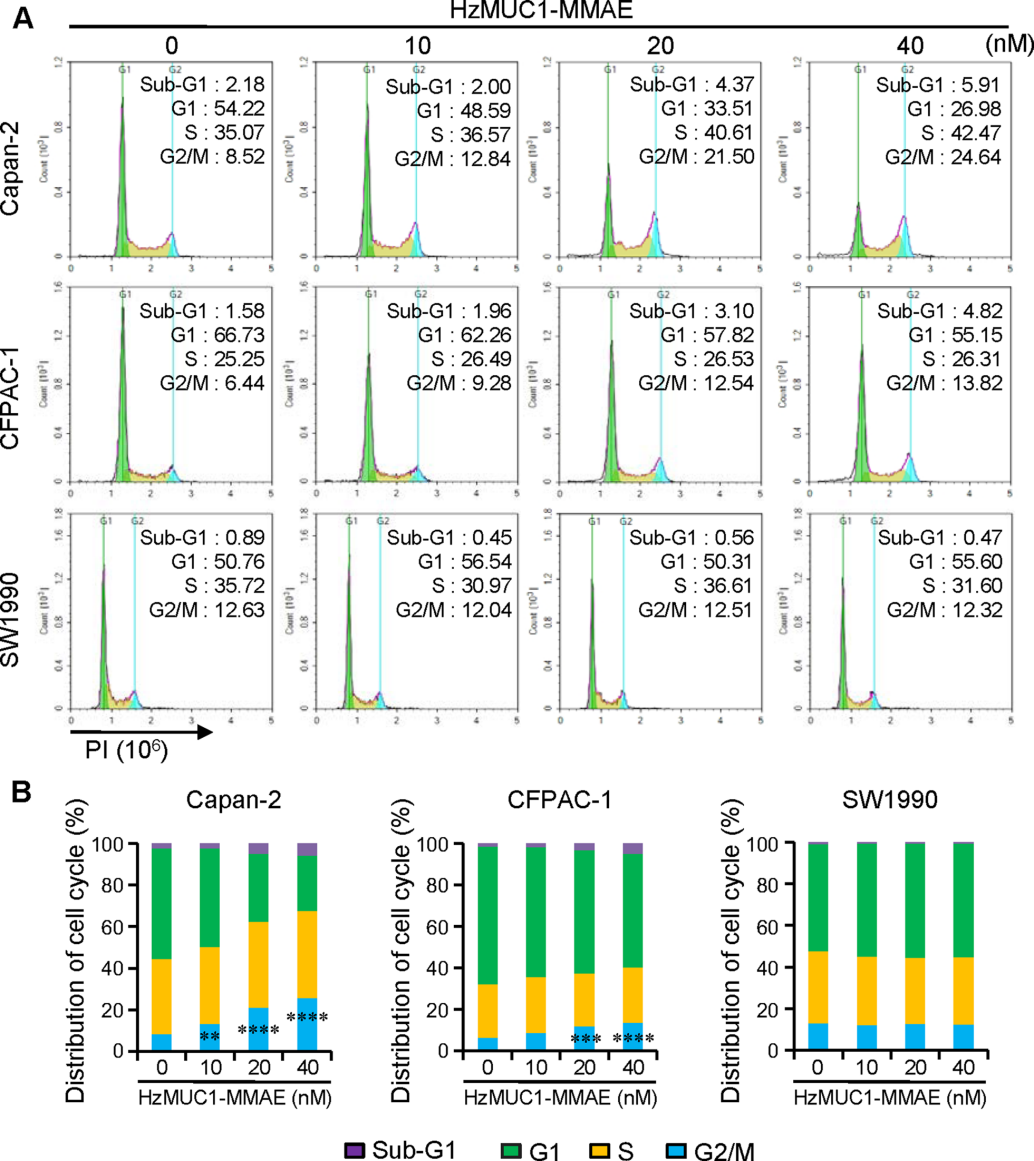
HzMUC1-MMAE induces cell cycle arrest at G2/M phase in pancreatic cancer cells. **A** Indicated pancreatic cell lines were treated with indicated concentrations of HzMUC1-MMAE for 24 h, fixed, and stained with Propidium iodide. G1, S, and G2/M phases of the cell cycle were analyzed by Flow cytometry using NovoExpress software. **B** Quantification of the flow cytometry analysis data from (**A**). The mean values from four different experiments were presented. The G2/M content of the HzMUC1-MMAE group was compared with that of vehicle control group. Statistical analyses were performed using One-way ANOVA with Bonferroni’s multiple comparision test, **P < 0.01, ***P < 0.001, ****P < 0.0001

To examine whether HzMUC1-MMAE affects cell cycle progression of pancreatic cancer cells, cells were treated the different concentrations of HzMUC1-MMAE (10, 20, 40 nM) for 24 h being subjected to DNA content analysis by flow cytometry (Fig. 3). As expected, HzMUC1-MMAE did not have any effect on cell cycle progression of MUC1 negative SW1990 cells. In contrast, HzMUC1-MMAE significantly increased the G2/M cell contents of Capan-2 and CFPAC-1 cells compared with cells treated with vehicle control (Fig. 3A, B). HzMUC1-MMAE increased the G2/M content in a dose dependent manner, from ~ 13 to 26% in Capan-2 cells and 9 to 14% in CFPAC-1 cells (Fig. 3B). Furthermore, HzMUC1-MMAE significantly increased the sub-G1 cell contents of Capan-2 and CFPAC-1 compared with cells treated with vehicle control (Fig. 3A, B). HzMUC1-MMAE increased the sub-G1 content in a dose dependent manner, from 2 to 6% in Capan-2 cells and 2 to 5% in CFPAC-1 cells (Fig. 3B). Our results indicate that HzMUC1-MMAE not only causes cell G2/M cycle arrest but also induces apoptosis of MUC1 positive pancreatic cancer cells.

### HzMUC1-MMAE enhances apoptosis of MUC1 positive pancreatic cancer cells

**Fig. 4:**
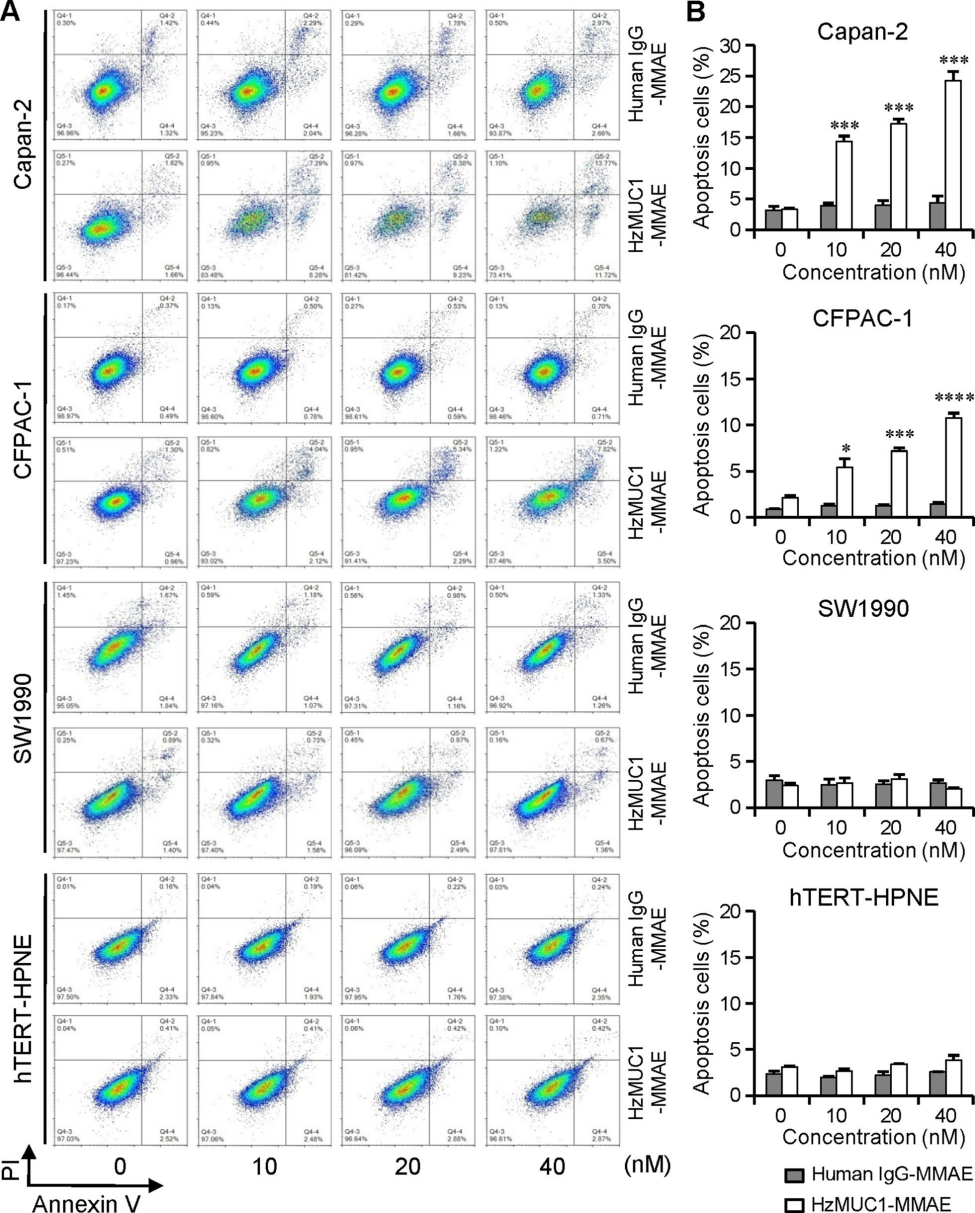
HzMUC1-MMAE induces apoptosis in pancreatic cancer cells. **A** The Capan-2, CFPAC-1, SW1990, and hTERT-HPNE cells were treated with HzMUC1-MMAE (10, 20, 40 nM) or human IgG-MMAE (10, 20, 40 nM) for 72 h. The cells were stained with annexin V and propidium iodide, and analyzed by flow cytometry using NovoExpress software. **B** Quantification of the flow cytometry analysis data from (**A**). The mean values from three different experiments were presented. HzMUC1-MMAE group was compared with control group. Statistical analyses were performed using One-way ANOVA with Bonferroni’s multiple comparision test, *P < 0.05, ***P < 0.001, ****P < 0.0001

To further provide evidence that HzMUC1-MMAE induces apoptosis of pancreatic cancer cells, cells were treated the human IgG-MMAE (10, 20, 40 nM) or HzMUC1-MMAE (10, 20, 40 nM) for 72 h and then stained with propidium iodide (cell death marker)and annexin V (early apoptosis marker) (Fig. 4). As an isotype control, human IgG-MMAE did not increase apoptosis of Capan-2, CFPAC-1, SW1990, and hTERT-HPNE cells. In contrast, HzMUC1-MMAE significantly enhanced apoptosis of Capan-2 and CFPAC-1 cells compared with control (Fig. 4A, B). HzMUC1-MMAE induced apoptosis in a dose dependent manner, from ~ 14 to 24% in Capan-2 cells and 5 to 11% in CFPAC-1 cells (Fig. 4B, P < 0.05). Furthermore, HzMUC1-MMAE did not increase apoptosis of MUC1 negative cells SW1990 and Normal pancreatic duct cells hTERT-HPNE. Our results further support that HzMUC1-MMAE significantly enhances apoptosis of MUC1 positive pancreatic cancer cells.

### HzMUC1-MMAE inhibits the growth of MUC1 positive pancreatic tumors in xenograft

**Fig. 5:**
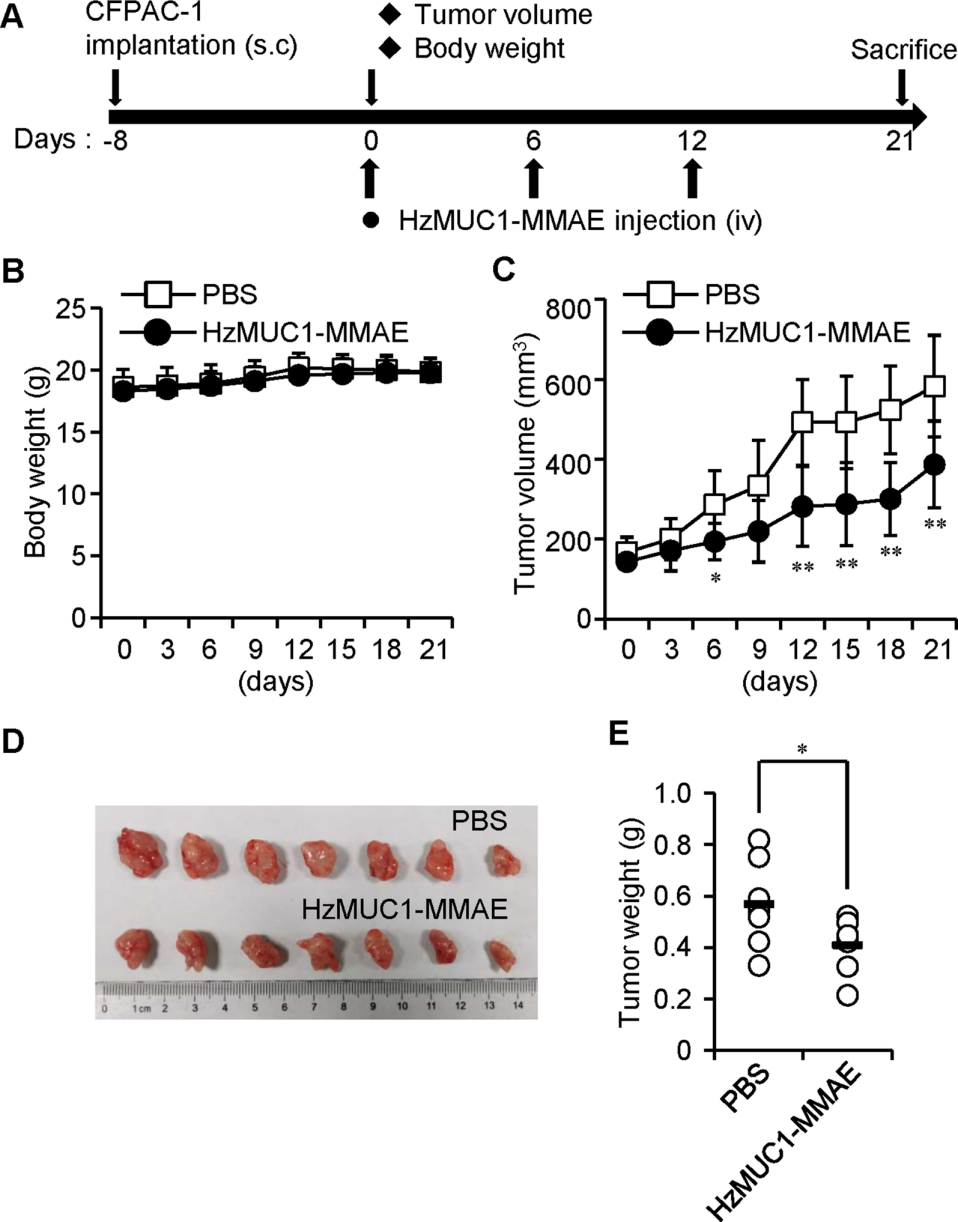
HzMUC1-MMAE impairs the growth of CFPAC-1 cell derived xenograft tumors. BALB/c nu/nu mice were subcutaneously injected with CFPAC-1 cells to induce tumor formation. Once the tumors reached 150 mm^3^ in volumes, the mice were randomized into two groups (seven mice/group) and treated with PBS (n = 7) or HzMUC1-MMAE (5 mg/kg, n = 7) by intravenous injection three times (once every 6-day). **A** HzMUC1-MMAE dosing scheme. **B** Body weights of mice for each treatment group. **C** The growth of tumor volumes over the experimental period. **D** Images of isolated tumors from mice euthanized at the end of experimental period. **E** Tumor weights for each treatment group. Statistical analyses were performed using unpaired two-tailed Student’s *t*-test. *P < 0.05, **P < 0.01, the HzMUC1-MMAE group was compared with that of PBS control group

To examine the efficacy of the HzMUC1-MMAE in decreasing pancreatic tumor growth in vivo, we utilized our established pancreatic tumor xenograft mouse model and evaluated the growth of tumors after injection of the ADC into mice. First, BALB/c nu/nu mice were subcutaneously injected with CFPAC-1 cells. When the CFPAC-1 tumors reached the sizes of 150 mm^3^, mice were randomized into two groups and treated with PBS and HzMUC1-MMAE (5 mg/kg). When assessing tumor volume and weight, we discovered that mice treated with the HzMUC1-MMAE displayed reduced growth of CFPAC-1 tumors compared to PBS-treated mice (Fig. 5C–E).

**Fig. 6:**
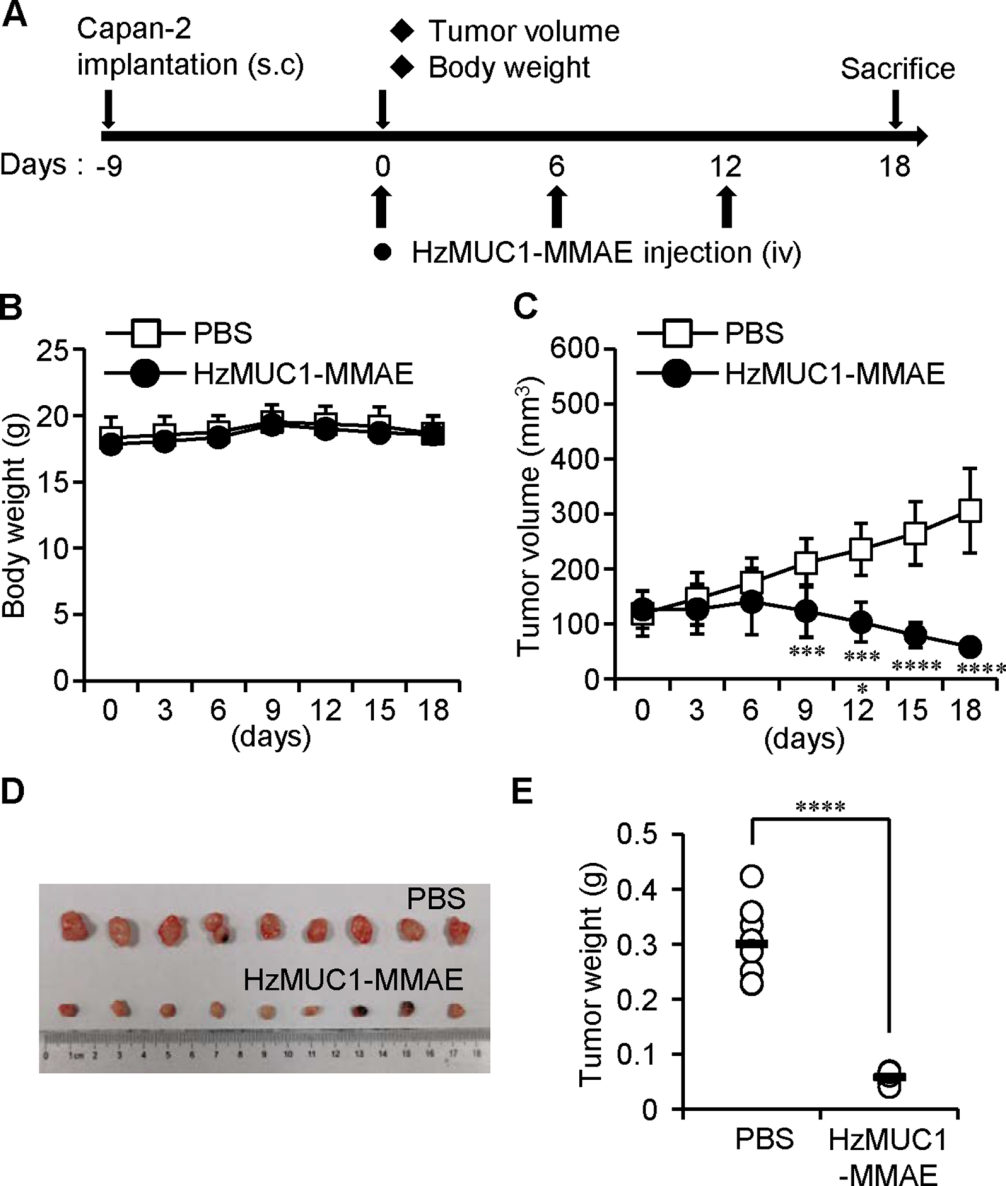
HzMUC1-MMAE dramatically inhibits the growth of Capan-2 xenograft tumor. BALB/c nu/nu mice were subcutaneously injected with Capan-2 cells to induce tumor formation. Once the tumors reached 120 mm^3^ in volumes, PBS (n = 9) or HzMUC1-MMAE (5 mg/kg, n = 9) was intravenous injected into mice three times (once every 6-day). **A** HzMUC1-MMAE dosing scheme. **B** Body weights for each treatment group. **C** The growth of tumor volumes over the experimental period. **D** Images of isolated tumors from mice euthanized at the end of experimental period. **E** Tumor weights for each treatment group. Statistical analyses were performed using unpaired two-tailed Student’s *t*-test. ***P < 0.001, ****P < 0.0001, the HzMUC1-MMAE group was compared with that of PBS control group

**Fig. 7:**
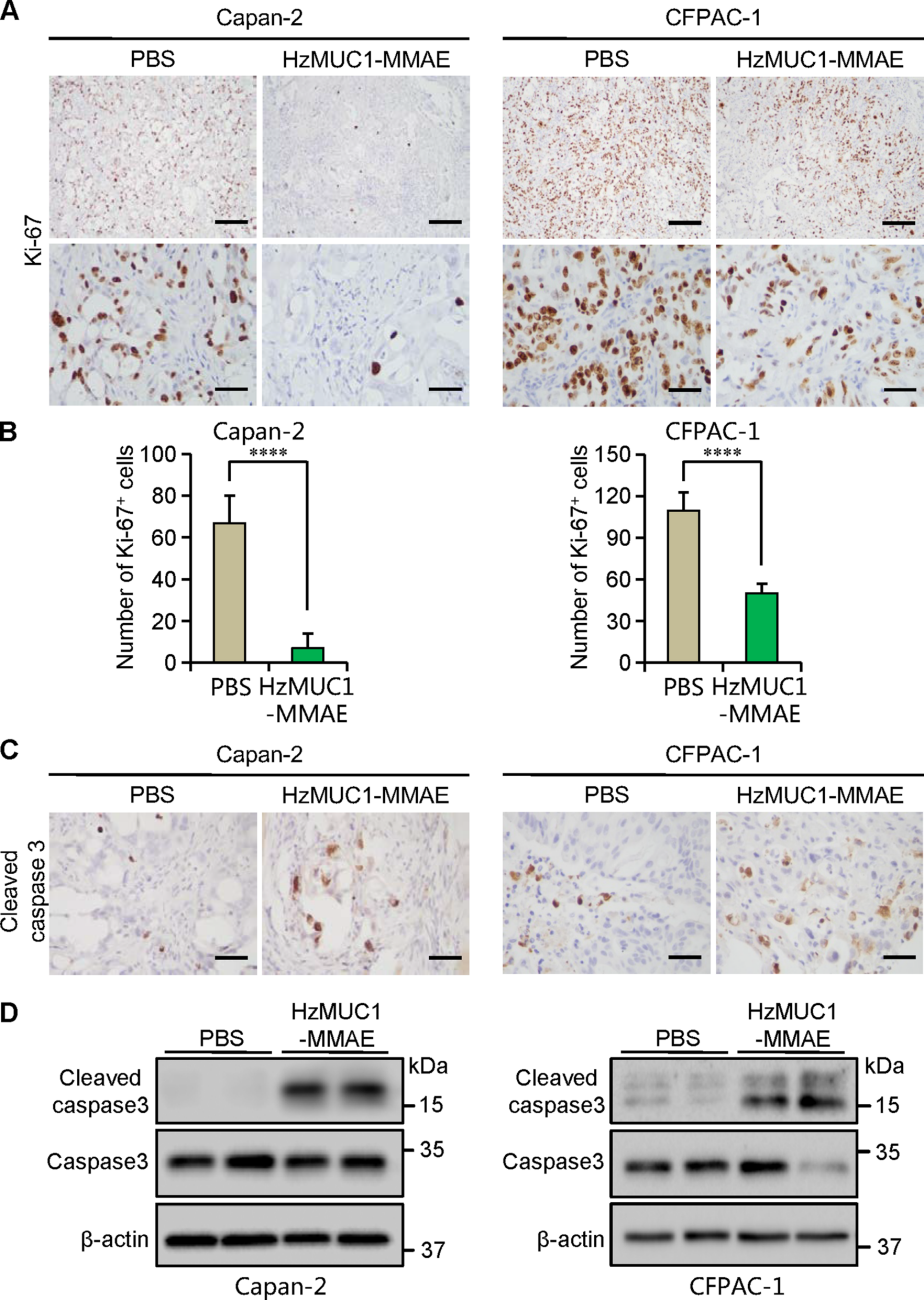
HzMUC1-MMAE inhibits the growth of Capan-2 and CFPAC-1 tumor by reducing cell proliferation and inducing apoptosis. Tumors dissected from mice treated with PBS or HzMUC1-MMAE (5 mg/kg) were sectioned, and subjected to immunohistochemistry staining with anti-Ki-67 (**A**) and anti-cleaved caspase-3 (**C**) antibodies respectively. Scale bar = 50 μm. **B** Quantification of the Ki-67 immunohistochemistry results. Ki-67 positive tumor cells were counted from five random 400x microscopic fields for each section. **D** Protein samples from Capan-2 and CFPAC-1 tumor tissues were resolved by SDS-PAGE and immunoblotted with anti-cleaved caspase 3 and anti-caspase 3 antibodies, and reprobed with anti-β-actin antibody as loading control. Statistical analyses were performed using unpaired two-tailed Student’s *t*-test. ****P < 0.0001, indicate statistical significance compared to PBS control group

Furthermore, BALB/c nu/nu mice were subcutaneously injected with Capan-2 cells. When the Capan-2 tumors reached the sizes of 120 mm^3^, the mice were intravenously injected through the tail vein with PBS and HzMUC1-MMAE. Compared to PBS treatment, treatment with HzMUC1-MMAE (5 mg/kg) dramatically inhibited the growth of Capan-2 xenograft tumors (Fig. 6C–E). The expression of MUC1 in Capan-2 and CFPAC-1 tumor tissue was examined by immunostaining with the anti-MUC1-CT antibody. The treatment of HzMUC1-MMAE does not affect MUC1 protein levels in xenograft tumor tissues (Additional file 1: Fig. S3). Ki-67 immunohistochemistry analysis revealed that the HzMUC1-MMAE treatment significantly reduced the proliferation of Capan-2 and CFPAC-1 tumor cells (Fig. 7A, B). Concurrently, the result of cleaved caspase-3 immunohistochemistry and immunoblot showed that HzMUC1-MMAE at the 5 mg/kg dose induced higher level of apoptosis compared with PBS in Capan-2 and CFPAC-1 tumors (Fig. 7C, D). Furthermore, the body weights of the mice were not affected by treatments with HzMUC1-MMAE, indicating that the antibody-drug conjugate had no notable side effects (Figs. 5B, 6B). Collectively, these experiments indicate that the efficacy of HzMUC1-MMAE against pancreatic tumors in xenograft models depends on the MUC1 level in pancreatic cancer cells.

## Discussion

Pancreatic cancer is a very challenging disease because most of the patients are already at the advanced stage before being diagnosed [22, 23]. Despite extensive efforts in searching for effective treatments for pancreatic cancer, there has been no major breakthrough for decades. The most difficult obstacle for the treatment of pancreatic cancer is the lack of symptoms for the disease at the early stage, and the late diagnosis of the disease at the advanced stage [23]. Another reason for the ineffective treatment of pancreatic cancer is that most of the standard therapeutics is consisted of the cytotoxic chemotherapy drugs, which have limited impact on overall survival [24, 25]. Therefore, there is an immediate need to develop effective targeted therapy for pancreatic cancer.

We previously developed the humanized MUC1 monoclonal antibody targeting MUC1. The HzMUC1 antibody targets the interaction region between MUC1-N and MUC1-C, unlike most current MUC1 antibodies that target variable tandem repeat region (VNTR) of MUC1-N [26]. MUC1-N subunit is frequently shed from tumor cells and present freely in the extracellular matrix and blood circulation in patients [27]. The free MUC1-N subunit could neutralize MUC1 therapeutic antibodies targeting MUC1-N. However, HzMUC1 antibody only binds the MUC1-N/MUC1-C heterodimer, which is present mainly on the surface of tumor cells, making it an ideal therapeutic antibody against MUC1 [21]. To address the clinical need for improved pancreatic cancer therapy, we here evaluated the potential utility of HzMUC1-MMAE ADCs in treating pancreatic cancer. The HzMUC1 antibody immunoprecipitated MUC1-N and MUC1-C from the pancreatic cancer cell lines Capan-2 and CFPAC-1 (Fig. 1B). This indicates the ability of HzMUC1 to bind the endogenous MUC1 in its native structure. In addition, confocal microscopy analysis of Capan-2 and CFPAC-1 cells clearly revealed that the HzMUC1 recognized MUC1 on the surface of pancreatic cancer cells (Fig. 1C). We determined that the HzMUC1 can specifically target MUC1 on the surface of pancreatic cancer cells.

The antibody is the main component of the antibody-drug conjugate. It should possess the target specificity and high binding affinity to the tumor antigens [28, 29]. In addition, the expression level of antigen on the surface of tumor cells will affect the antitumor activity of ADC [30–33]. Under normal physiological conditions, MUC1-N and MUC1-C heterodimers are formed through non-covalent bonds and are localized in the plasma membrane. However, this heterodimer can be dissociated under the stimulation of proinflammatory cytokines. Interferon-γ and TNF-α enhance the activity of extracellular integrin metalloproteinase, and the catalysis of these enzymes leads to the shedding of MUC1-N from the cell surface. However, the molecular mechanism of how MUC1-N shed from the cell surface is unclear [34, 35]. It has been reported that MUC1 protein is expressed at high levels over the entire surface of tumor cells from diverse types [8]. Its isoforms consist of the full-length type containing SEA domain, and MUC1-C type [8]. However, the expression level of each MUC1 isoform on the surface of cancer cells is unclear, especially in pancreatic cancer. Therefore, we emphasize here that the HzMUC1 antibody recognizes a MUC1 isoform, which contains SEA domain, expressed on the surface of pancreatic cancer cells. Importantly, we found that the conjugate of HzMUC1 and MMAE as ADC is very effective in killing MUC1 positive pancreatic cancer cells at low concentration (Fig. 2). We also found that HzMUC1-MMAE treatment could effectively inhibit the growth of established Capan-2 xenograft tumor in BALB/c nu/nu mice without weight loss or other obvious toxicity (Fig. 6).

Interestingly, our results revealed that HzMUC1-MMAE is less effective in inhibiting the growth of CFPAC-1 tumors compared with that of Capan-2 tumors (Figs. 5, 6). One possibility is that MUC1 is hyperglycosylated in the region that is recognized by HzMUC1 in CFPAC-1 cells, which prevents HzMUC1-MMAE from binding the cell surface MUC1 and killing CFPAC-1 cells in vivo. There are many O-linked and five predicted N-linked glycosylation sites in the extracellular domain of MUC1 protein [36], in which two of predicted N-linked glycosylation sites are potentially located in the interaction region between MUC1-N and MUC1-C. Consistent with this hypothesis, immunostaining using HzMUC1 showed that MUC1 fluorescent signal on the cell surface is much lower in CFPAC-1 cells compared with in Capan2 (Fig. 1C). Published study also demonstrated that N-linked glycosylation of PD-L1 decreases anti-PD-L1 antibody binding affinity to PD-L1 and interferes accurate detection of PD-L1 level in human cancer cells [37]. The second possibility is that the decreased affinity of HzMUC1 to human MUC1, after humanization of mouse anti-MUC1 SEA antibody through modification by CDR grafting technology (data no shown), contributes to the reduced efficacy of HzMUC1 against CFPAC-1 tumors in vivo. In the future, we plan to improve the binding affinity of HzMUC1 to MUC1 by screening the human Ig phage display library using the variable region of the mouse anti-MUC1 SEA antibody as the template and the MUC1 SEA domain region as the antigen, which should help generate higher affinity humanized antibodies that mimic the variable region of the mouse monoclonal antibody and bind to the same antigen determinant as described [38].

ADC is a monoclonal antibody conjugated with a potent cytotoxic drug through appropriate links. It has been proved to be an effective targeted drug (such as T-DM1) for the treatment of HER2 positive breast cancer [35]. MMAE is a synthetic derivative of dolastatin 10, which inhibits mitosis by inhibiting tubulin polymerization. MMAE as an payloads of ADC widely used in clinical trials and clinical practice, and has reliable efficacy and safety [39]. Our data show that HzMUC1-MMAE treatment could induce the G2/M cell cycle arrest and apoptosis in the MUC1 positive pancreatic cancer cells Capan-2 and CFPAC-1 (Figs. 3, 4).

In conclusion, our HzMUC1-MMAE ADC significantly inhibits the growth of MUC1 positive pancreatic cancer cells in vitro and in vivo. The HzMUC1 ADC is a potentially effective antibody drug for the treatment of pancreatic cancer.

## Supplementary Information

**Additional file 1.** Additional materials and methods. **Figure S1.** MMAE inhibits the growth of pancreatic cancer cells. **Figure S2.** Effect of Mc-vc-PABC linker on growth of pancreatic cancer cells. **Figure S3.** Expression of the MUC1 protein in Capan-2 and CFPAC-1 xenograft tumor tissues.

## Data Availability

All datasets obtained and analyzed during this study are available from the corresponding author on reasonable request.

## References

[1] Siegel RL, Miller KD, Jemal A (2017). Cancer Statistics. Cancer J Clin..

[2] Vincent A, Herman J, Schulick R, Hruban RH, Goggins M (2011). Pancreatic cancer. Lancet.

[3] Levitin F, Stern O, Weiss M, Gil-Henn C, Ziv R, Prokocimer Z (2005). The MUC1 SEA module is a self-cleaving domain. J Biol Chem.

[4] Hattrup CL, Gendler SJ (2008). Structure and function of the cell surface (tethered) mucins. Annu Rev Physiol.

[5] Gendler SJ (2001). MUC1, the renaissance molecule. J Mammary Gland Biol Neoplasia.

[6] Yolken RH, Peterson JA, Vonderfecht SL, Fouts ET, Midthun K, Newburg DS (1992). Human milk mucin inhibits rotavirus replication and prevents experimental gastroenteritis. J Clin Invest.

[7] Schroten H, Hanisch FG, Plogmann R, Hacker J, Uhlenbruck G, Nobis-Bosch R (1992). Inhibition of adhesion of S-fimbriated Escherichia coli to buccal epithelial cells by human milk fat globule membrane components: a novel aspect of the protective function of mucins in the nonimmunoglobulin fraction. Infect Immun.

[8] Kufe DW (2009). Mucins in cancer: function, prognosis and therapy. Nat Rev Cancer.

[9] Lau SK, Weiss LM, Chu PG (2004). Differential expression of MUC1, MUC2, and MUC5AC in carcinomas of various sites: an immunohistochemical study. Am J Clin Pathol.

[10] Striefler JK, Riess H, Lohneis P, Bischoff S, Kurreck A, Modest DP (2021). Mucin-1 protein is a prognostic marker for pancreatic ductal adenocarcinoma: results from the CONKO-001 study. Front Oncol.

[11] Bose M, Sanders A, De C, Zhou R, Lala P, Shwartz S (2022). Targeting tumor-associated MUC1 overcomes anoikis-resistance in pancreatic cancer. Transl Res..

[12] Sahraei M, Roy LD, Curry JM, Teresa TL, Nath S, Besmer D (2012). MUC1 regulates PDGFA expression during pancreatic cancer progression. Oncogene.

[13] Syrkina MS, Rubtsov MA (2019). MUC1 in Cancer Immunotherapy - New Hope or Phantom Menace?. Biochem (Mosc).

[14] Zhou R, Yazdanifar M, Roy LD, Whilding LM, Gavrill A, Maher J (2019). CAR T cells targeting the Tumor MUC1 glycoprotein reduce triple-negative breast Cancer Growth. Front Immunol.

[15] You F, Jiang L, Zhang B, Lu Q, Zhou Q, Liao X (2016). Phase 1 clinical trial demonstrated that MUC1 positive metastatic seminal vesicle cancer can be effectively eradicated by modified Anti-MUC1 chimeric antigen receptor transduced T cells. Sci China Life Sci.

[16] Wilkie S, Picco G, Foster J, Davies DM, Julien S, Cooper L (2008). Retargeting of human T cells to tumor-associated MUC1: the evolution of a chimeric antigen receptor. J Immunol.

[17] Posey AD, Schwab RD, Boesteanu AC, Steentoft C, Mandel U, Engels B (2016). Engineered CAR T cells targeting the Cancer-Associated Tn-Glycoform of the membrane mucin MUC1 control Adenocarcinoma. Immunity.

[18] Kufe DW (2013). MUC1-C oncoprotein as a target in breast cancer: activation of signaling pathways and therapeutic approaches. Oncogene.

[19] Pichinuk E, Chalik M, Benhar I, Ginat-Koton R, Ziv R, Smorodinsky NI (2020). In vivo anti-MUC1(+) tumor activity and sequences of high-affinity anti-MUC1-SEA antibodies. Cancer Immunol Immunother.

[20] Wu G, Maharjan S, Kim D, Kim JN, Park BK, Koh H (2018). A Novel Monoclonal Antibody Targets Mucin1 and Attenuates Growth in Pancreatic Cancer Model. Int J Mol Sci..

[21] Wu G, Li L, Qiu Y, Sun W, Ren T, Lv Y (2021). A novel humanized MUC1 antibody-drug conjugate for the treatment of trastuzumab-resistant breast cancer. Acta Biochim Biophys Sin (Shanghai).

[22] Siegel RL, Miller KD, Fuchs HE, Jemal A (2021). Cancer Statistics. Cancer J Clin..

[23] Nai Q, Luo H, Zhang P, Hossain MA, Gu P, Sidhom IW (2015). How early can pancreatic cancer be recognized? A case report and review of the literature. Case Rep Oncol.

[24] Cartwright T, Richards DA, Boehm KA (2008). Cancer of the pancreas: are we making progress? A review of studies in the US Oncology Research Network. Cancer Control.

[25] Lockhart AC, Rothenberg ML, Berlin JD (2005). Treatment for pancreatic cancer: current therapy and continued progress. Gastroenterology.

[26] Bose M, Mukherjee P (2020). Potential of Anti-MUC1 antibodies as a targeted therapy for gastrointestinal cancers. Vaccines (Basel)..

[27] Moreno M, Bontkes HJ, Scheper RJ, Kenemans P, Verheijen RH, von Mensdorff-Pouilly S (2007). High level of MUC1 in serum of ovarian and breast cancer patients inhibits huHMFG-1 dependent cell-mediated cytotoxicity (ADCC). Cancer Lett.

[28] Peters C, Brown S (2015). Antibody-drug conjugates as novel anti-cancer chemotherapeutics. Biosci Rep..

[29] Hughes B (2010). Antibody-drug conjugates for cancer: poised to deliver?. Nat Rev Drug Discov.

[30] Damelin M, Zhong W, Myers J, Sapra P (2015). Evolving strategies for target selection for antibody-drug conjugates. Pharm Res.

[31] Diamantis N, Banerji U (2016). Antibody-drug conjugates–an emerging class of cancer treatment. Br J Cancer.

[32] Tipton TR, Roghanian A, Oldham RJ, Carter MJ, Cox KL, Mockridge CI (2015). Antigenic modulation limits the effector cell mechanisms employed by type I anti-CD20 monoclonal antibodies. Blood.

[33] Khongorzul P, Ling CJ, Khan FU, Ihsan AU, Zhang J (2020). Antibody-drug conjugates: a Comprehensive Review. Mol Cancer Res.

[34] Thathiah A, Carson DD (2004). MT1-MMP mediates MUC1 shedding independent of TACE/ADAM17. Biochem J.

[35] Martinez MT, Perez-Fidalgo JA, Martin-Martorell P, Cejalvo JM, Pons V, Bermejo B (2016). Treatment of HER2 positive advanced breast cancer with T-DM1: a review of the literature. Crit Rev Oncol Hematol.

[36] Nath S, Mukherjee P (2014). MUC1: a multifaceted oncoprotein with a key role in cancer progression. Trends Mol Med.

[37] Lee HH, Wang YN, Xia W, Chen CH, Rau KM, Ye L (2019). Removal of N-Linked glycosylation enhances PD-L1 detection and predicts Anti-PD-1/PD-L1 therapeutic efficacy. Cancer Cell.

[38] Jespers LS, Roberts A, Mahler SM, Winter G, Hoogenboom HR (1994). Guiding the selection of human antibodies from phage display repertoires to a single epitope of an antigen. Biotechnol (N Y).

[39] Doronina SO, Toki BE, Torgov MY, Mendelsohn BA, Cerveny CG, Chace DF (2003). Development of potent monoclonal antibody auristatin conjugates for cancer therapy. Nat Biotechnol.

