# Supplementary material for: Therapeutic effect of a MUC1-specific monoclonal antibody-drug conjugates against pancreatic cancer model
Source: Cancer Cell Int. 2022 Dec 27;22:417. doi: 10.1186/s12935-022-02839-w (PMC9793597; doi:10.1186/s12935-022-02839-w)
Supplement: Supplementary file 1 — Additional file 1. Additional materials and methods. Figure S1. MMAE inhibits the growth of pancreatic cancer cells. Figure S2. Effect of Mc-vc-PABC linker on growth of pancreatic cancer cells. Figure S3. Expression of the MUC1 protein in Capan-2 and CFPAC-1 xenograft tumor tissues. [file 12935_2022_2839_MOESM1_ESM.doc]

**Therapeutic effect of a MUC1-specific monoclonal antibody-drug conjugates against pancreatic cancer model**

Guang Wu^1, †, *^, Lan Li^2, †^, Mengnan Liu^1, †^, Chunyan Chen^1^, Guangze Wang^1^, Zewei Jiang^1^, Yaqian Qin^3^, Licai He^1^, Hongzhi Li^1^, Jiawei Cao^1^, Haihua Gu^1,*^

^1^Key Laboratory of Laboratory Medicine, Ministry of Education, School of Laboratory Medicine and Life Sciences, and ^2^School of Public Health and Management, Wenzhou Medical University, Wenzhou 325035, China. ^3^Medical Research Center, the First Affiliated Hospital of Wenzhou Medical University, Wenzhou 325000, China.

† Equal contribution to this work

* Corresponding authors: Guang Wu, E‑mail: guangwu@wmu.edu.cn. Haihua Gu, E-mail: haihuagu@wmu.edu.cn.

**Additional materials and methods**

Colony formation assay

Cells were seeded in 48-well tissue culture plates, and treated with the indicated concentrations of MMAE (MCE, Monmouth, NJ, USA), Mc-Val-Cit-PABC-PNP (a drug-linker agent, MCE). After 5 days, colonies were fixed with 4% paraformaldehyde solution, and stained with 0.5% crystal violet solution at RT for 20 min. Stained crystal violet dye was solubilized with 10% acetic acid. Absorbance was measured at 540 nm using a microplate reader (Molecular Devices, San Jose, CA). Cell growth curve was measured using Prism (Graph Pad Software Inc). The inhibitory concentration (IC_50_) for MMAE was determined using SPSS statistics software.

**Additional Figure 1**


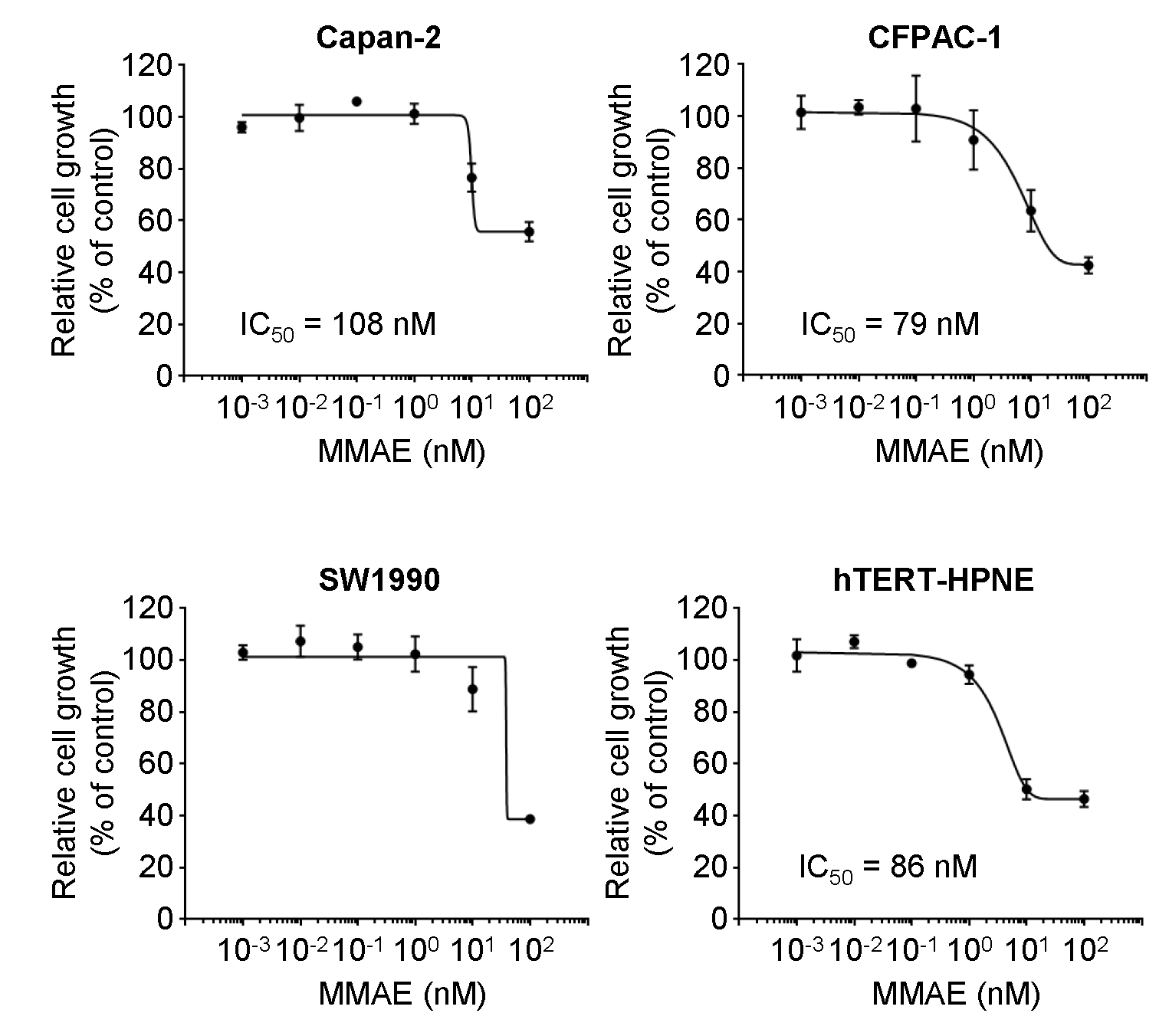


**Figure S1. MMAE inhibits the growth of pancreatic cancer cells.** Capan-2, CFPAC-1, SW1990 and HPNE cells were treated with indicated concentrations of MMAE. Cell growth was determined by staining the plates with crystal violet and quantified by OD 540 nm absorbance. Percent of relative cell growth was shown as the non MMAE treatment was set to 100. The IC_50_ values are indicated. The results shown are representative of data from 3 independent experiments.

**Additional Figure 2**


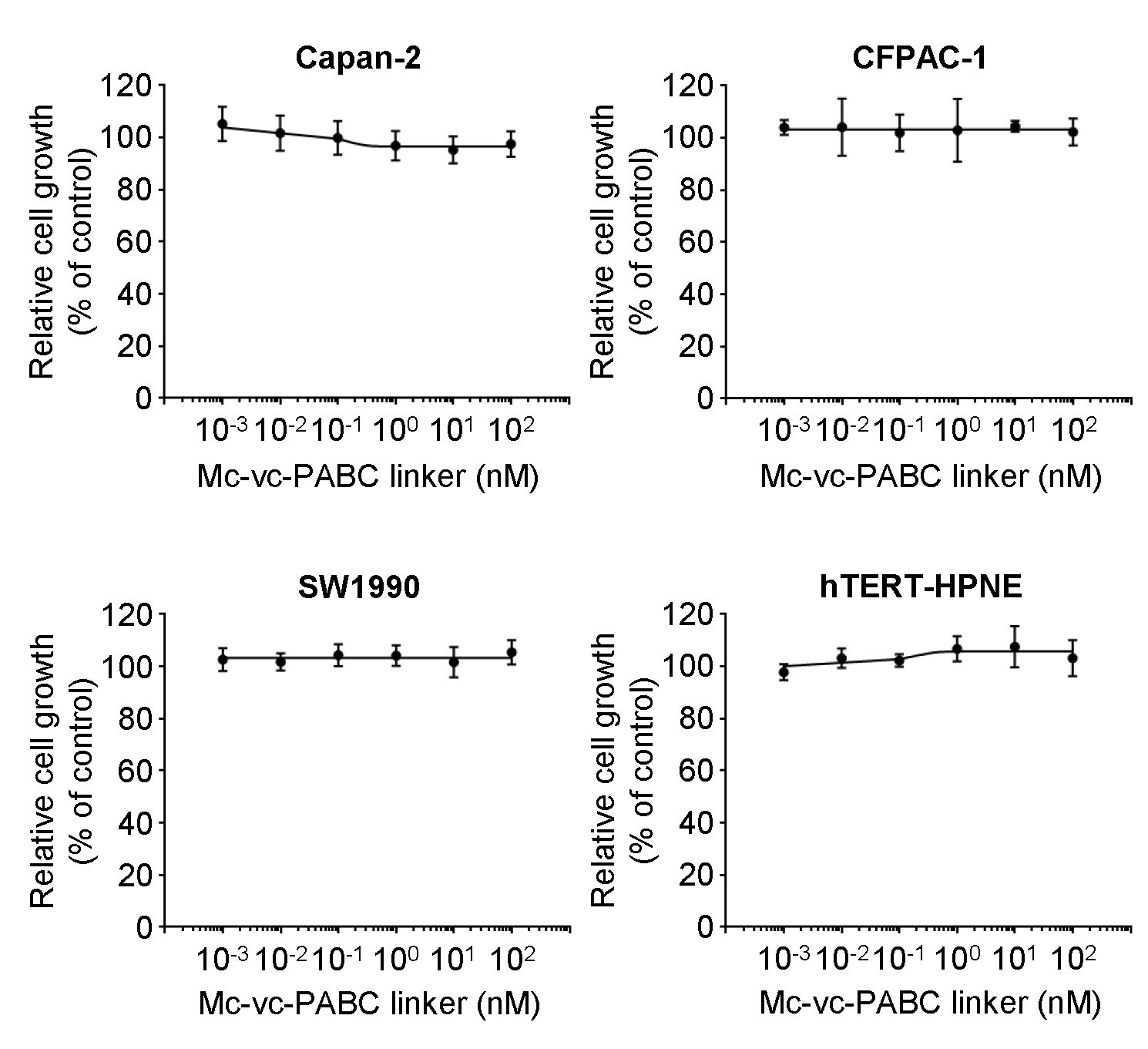


**Figure S2. Effect of Mc-vc-PABC linker on growth of pancreatic cancer cells.** Capan-2, CFPAC-1, SW1990 and HPNE cells were treated with indicated concentrations of Mc-vc-PABC linker for 5 days. Cell growth was determined by staining the plates with crystal violet and quantified by OD 540 nm absorbance. Percent of relative cell growth was shown as the non Mc-vc-PABC linker treatment was set to 100.

**Additional Figure 3**

**
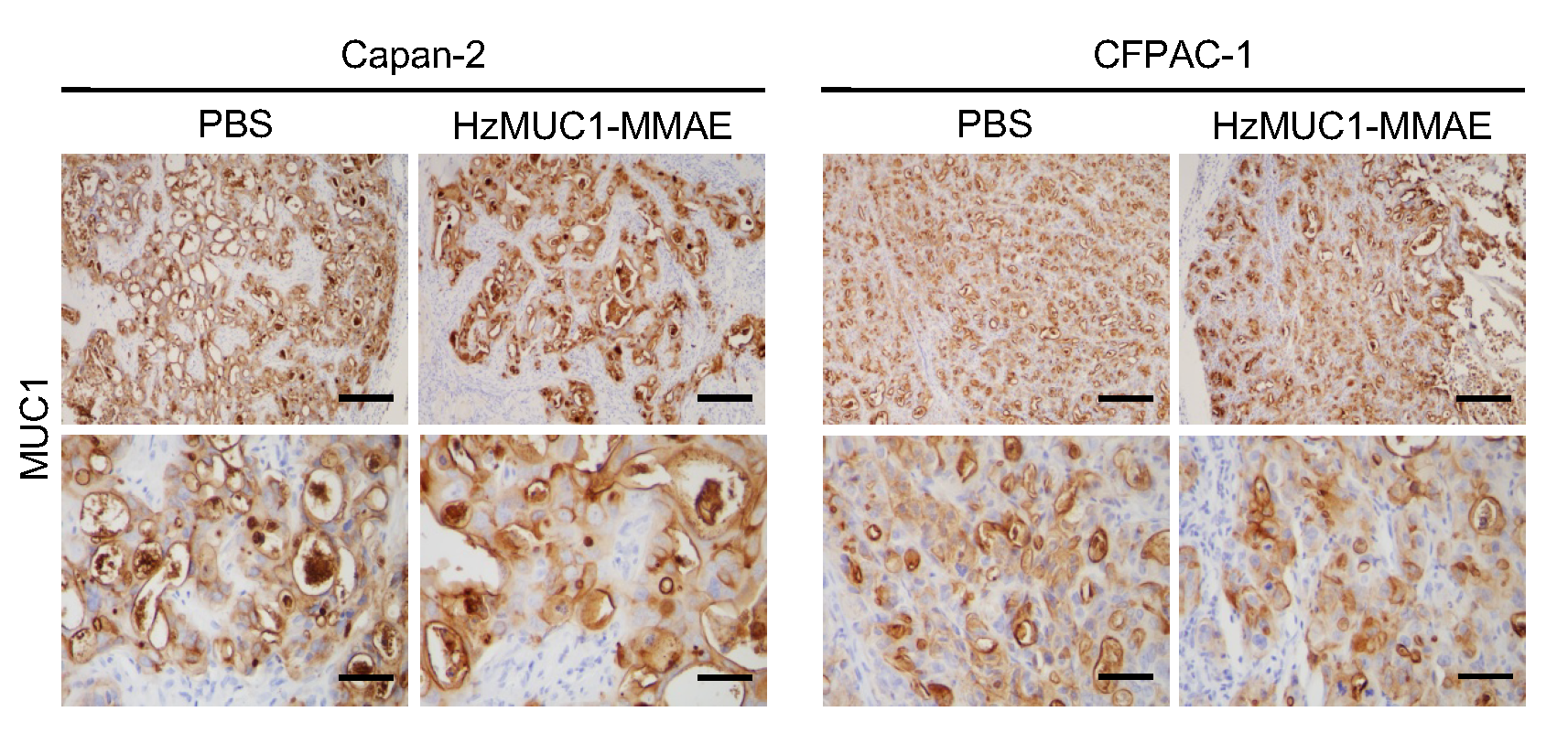
**

Figure S3. Expression of the MUC1 protein in Capan-2 and CFPAC-1 xenograft tumor tissues. Tumors dissected from mice treated with PBS or HzMUC1-MMAE (5 mg/kg) were sectioned, and subjected to immunohistochemistry staining with anti-MUC1-CT antibody. Scale bar =50 μm.
